# Haptic-Based Real-Time Platform for Microswarm Steering in a Multi-Bifurcation Vascular Network

**DOI:** 10.3390/nano14231917

**Published:** 2024-11-28

**Authors:** Benjamin W. Jarvis, Kiana Abolfathi, Riccardo Poli, Ali Kafash Hoshiar

**Affiliations:** School of Computer Science and Electronic Engineering, University of Essex, Colchester CO4 3SQ, UK; bj20907@essex.ac.uk (B.W.J.); kiana.abolfathi@essex.ac.uk (K.A.); rpoli@essex.ac.uk (R.P.)

**Keywords:** microrobotics, haptic-based guidance, electromagnetic actuation, microswarm

## Abstract

The use of electromagnetic fields to control a collection of magnetic nanoparticles, known as a microswarm, has many promising applications. Current research often makes use of accurate but time-consuming simulations lacking real-time human input. On the contrary, human interaction is possible with a real-time simulator, allowing the collection of valuable user interaction data. This paper presents the development and validation of a real-time two-dimensional microswarm simulator to accommodate the human interaction aspect. A haptic device is used to steer the microswarm through a multi-bifurcation vascular network towards a selected outlet. The percentage of particles reaching the selected outlet is used as the success metric. The simulator is verified against collected real-world experimental data and shows an 8% deviation. Parametric studies demonstrate the most influential parameters. We found that reducing the magnetic gradient from 1000 mT/m to 100 mT/m resulted in a decrease in recorded performance from 100% to 30.8%. Variation in fluid flow also had a considerable effect on the recorded performance, presenting a drop from 100% to 35.3% when fluid flow velocities increased from 0.005 m/s to 0.06 m/s. Changing the starting arrangement of particles resulted in a drop to 59% over the same range of fluid flow velocities.

## 1. Introduction

**Figure 1:**
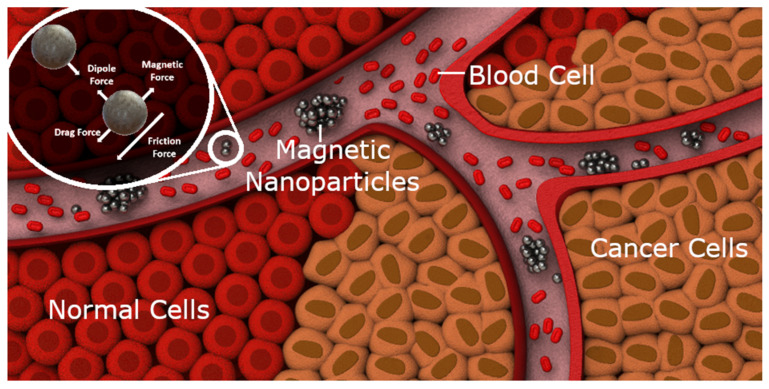
Illustrative representation of a conceptual microswarm application. The illustrated microswarm is guided within a vascular network for the purpose of cancer treatment, an example of a target application for this technology. Superimposed on the top-left corner is a free-body diagram of the major forces acting upon the particles.

The miniaturization of medical devices significantly enhances surgical outcomes and minimizes surgical side effects [1,2]. Medical microrobotics involves the design, fabrication, and operation of robots that are extremely miniaturized, often at the scale of micrometers. These microrobots can be broadly categorized as tethered (individual), untethered (individual), or as microswarms [3,4]. A microswarm is a controlled collection of micro-objects, typically magnetic micro/nanoparticles [5,6,7]. Figure 1 displays a conceptual image demonstrating cancer treatment as an example future application of medical microswarms, with other uses such as targeted drug delivery [8,9,10] and hyperthermia treatment [11]. Other technologies can also be used for cell manipulation [12].

Existing helical-type microrobotics demonstrate a nonreciprocal movement [3,7,13]. A magnetic torque is applied that allows the helical drive to pull or push the microrobot through the fluid [14], similar to the mechanics of a bacterial flagella [15,16]. Magnetic torque can also be used in the aggregation and steering of microswarms [4,17].

Magnetically actuated microswarms show promise in several medical applications, including localized hyperthermia treatment [9,18] and targeted drug delivery [19].

Microswarms are steered by an electromagnetic force or torque [20,21]. The successful steering of a microswarm, however, depends on achieving reliable and precise guidance [22]. Successful microswarm steering is difficult and, thus, requires computational modelling. Simulation platforms provide an invaluable testbed to experiment with new techniques [17,20], facilitating easier access to investigate user control strategies. Simulation platforms also allow for haptic control integration [23], where kinematic feedback can be given to the user, for example, in the form of restricting the user’s movement in a certain area or pushing against the user’s input [23].

Improving the accuracy of these simulators is computationally expensive, making the integration of real-time human-in-the-loop control challenging. Human-based guidance is important as it facilitates improved operator control of any actions being performed using robotics.

Haptic interaction allows for information transfer between a robot and a human user. Existing haptic-based control approaches use invisible walls [23] (restrictions to the user’s movement in certain directions) to guide the user. One-dimensional magnetic guidance for microswarms has been introduced [24,25], with recent advancements regarding multi-dimensional control [23].

This paper builds upon a real-time two-dimensional haptic-based simulator first introduced by Jarvis et al. [26]. The simulator is MATLAB-based (version 2023a), runs in real time, and integrates a Novint Falcon haptic device to facilitate real-time user interaction. A fluid flow model obtained through COMSOL simulation provides realistic fluid flow. The results are verified against experimental data, collected using a Magnebotix MFG-100-i multi-direction electromagnet system. This work presents a digital shadow platform designed for the multi-directional magnetic-field steering of a microswarm. The real-time, haptic-based platform facilitates microswarm steering studies and has the potential to be extended to 3D applications.

The remainder of this paper is organized as follows. Section 2.1 introduces the design concept for the platform. Section 2.2 details the modelling approach adopted. Section 2.3 presents the validation processes. Section 3 reports on the parametric study we conducted. Finally, Section 4 provides the conclusions.

## 2. Materials and Methods

### 2.1. Design and Implementation

**Figure 2:**
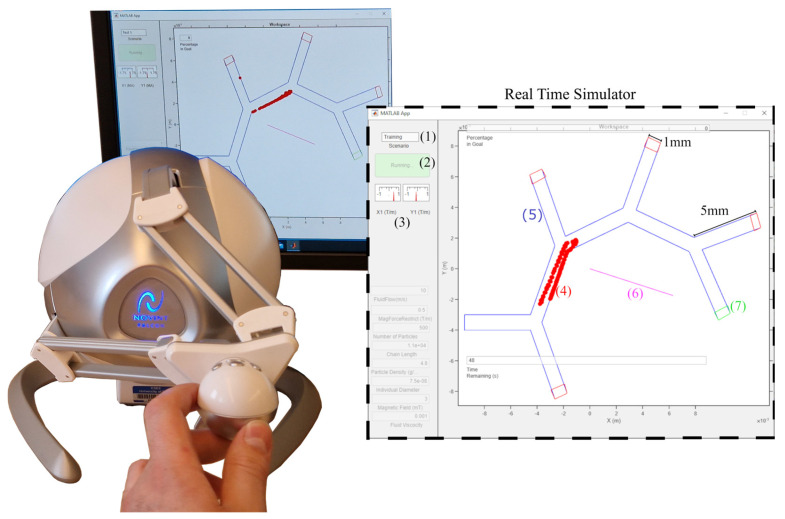
Setup of simulator including haptic device. The different sections of the simulator are as follows: (1) the current test number, (2) a start/stop button, (3) a selection of dials to show the magnetic gradient input provided by the user, (4) particles within the simulated vascular network, (5) the simulated vascular network comprising an outline of navigable multi-bifurcations (all vessels have a width of 1 mm and a length of 5 mm), (6) magenta line displaying the direction and magnitude of the magnetic gradient input from the user, and (7) the green goal outlet.

The setup for the simulator is shown in Figure 2. As a testbed for the proposed platform, a multi-bifurcation vascular network with one input and five outputs is constructed in the simulator. The simulator displays a top–down, two-dimensional visualization of particle locations, presented to the user in a simplified, planar format. In this study, the third dimension is not incorporated into the model. Figure 2 demonstrates some other features of the simulator: the magenta line originating from the centre of the workspace represents the direction and relative magnitude of user input. The current multi-bifurcation to navigate is presented in blue, and the goal outlet in green. User controls such as a start button and information boxes are present. The simulator is used to study whether the magnetically controlled microswarm can be externally guided to the marked goal outlet.

**Figure 3:**
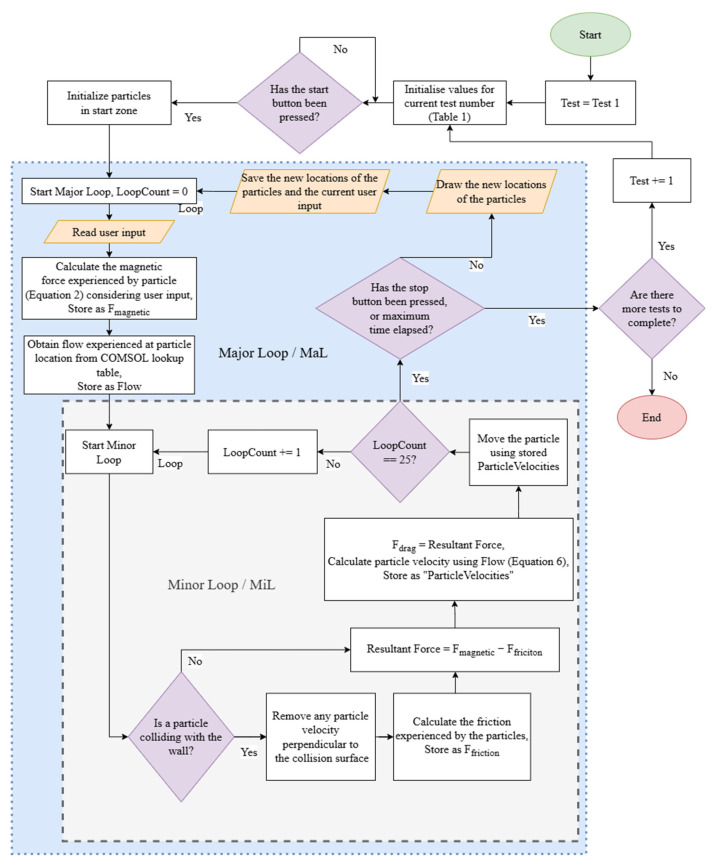
Flow chart showing the design of the real-time simulator, including content of the MaL and MiL.

Figure 3 describes the simulation implementation as a flow chart. To improve performance, the simulation code is split into two loops: the major loop (MaL) and the minor loop (MiL). The MiL is repeated 25 times in each MaL, as shown in Figure 3. The MiL implements the mathematical model that will be introduced in Section 2.2. The MaL handles reading of the user input, providing the haptic feedback using the Novint Falcon haptic device if applicable, and storing both particle locations and user input data in the log file. The user inputs the magnetic gradient within the MaL, which is then passed to the MiL to be used in the mathematical model. The fluid velocity used in the mathematical model is also determined in the MaL. The MaL subroutine is started every 0.05 s by a timer initialized in the user interface (UI) class. The MiL is implemented as a for loop within the MaL subroutine.

Within the MiL, a collision detection method is invoked. The collision detection method checks using the inbuilt MATLAB function ‘inpolygon’, upon moving the particles, if any of them have exited the multi-bifurcation model boundary. This collision model is extended using polygons to define further boundary conditions. Any particles outside the model boundary are reversed along their trajectory, then moved along a new reflected trajectory as though they bounced off the wall.

Due to the unconscious inference properties of human vision [27], the simulator provides enough visual information to users to experience a smooth particle motion without needing to update the UI as frequently as the 20 Hz used in the MaL. This allows the simulator to maximize time spent on model computation by updating aspects of the UI, such as particle locations, with a separate subroutine that runs less frequently than the MaL subroutine.

The simulator is paired with a Novint Falcon (Albuquerque, NM, USA) haptic device, as seen in Figure 2, to obtain user input and provide a kinaesthetic haptic response. The Novint Falcon runs using a compiled C++ code as a function call from MATLAB, allowing for direct access to variable outputs and inputs. The chosen default behaviour of the haptic feedback is a spring force of a maximum between 3 and 4 N that pulls the device back to a neutral rest position and includes all of the available (approximately) 10 cm × 10 cm workspace for input. The z axis (towards and away from the user) is not recorded.

### 2.2. Mathematical Model for the Steering Platform

Equation (1) represents the major forces acting during the magnetic steering process. For simplicity, without the loss of generality, forces such as gravity and buoyancy are excluded, instead implementing a two-dimensional simulator with a top–down view (shown in Figure 2).

(1) $$\begin{array}{c} m_{i}\frac{dv_{i}}{dt}=F_{magnetic}+F_{drag}+F_{friction}+F_{other} \end{array}$$

Equation (1) outlines the governing Newtonian dynamics of magnetic nanoparticle steering. In this approach, the velocity and location of particles, as well as all acting forces, are presumed constant for the duration of each MiL iteration. This facilitates a manageable computational load while still approximating real-world system behaviour. Through our parametric studies, we determined that the chosen integration timestep for the MiL, 0.05/25 = 0.002 s, provides an optimal balance of accuracy and performance. Other default values for the parameters used are listed in Table 1. The magnetic force is the only input force to steer the microswarm in real time. This is defined as

(2) $$\begin{array}{c} F_{magnetic}=V_{eq}\mu_{1}M_{m}\rho \nabla H \end{array}$$

where *V_eq_* is the volume of the particle chain, *µ*_1_ = *µ*_0_ is the vacuum permeability constant, *M_m_* is the magnetization per unit mass (magnetization for brevity), *ρ* is the particle density, and ∇***H*** is the magnetic gradient.

To convert magnetization per unit mass Mm to SI units it must be multiplied by the material density *ρ* [28]. The particle chain is assumed to be a cylinder, but in the simulation is modelled as a sphere of equivalent volume to the cylinder. The particle chain cylinder has a diameter *d* (particle diameter) and height *nd* (*n* being the number of particles in the chain), so the diameter of a volume-equivalent sphere is

(3) $$\begin{array}{c} D_{eq}=d\sqrt[{3}]{3n} \end{array}$$

where *d* is the individual particle diameter. The simulator should still provide a valid model when altering variables such as particle diameter, provided other parameters are altered to match the properties of the particles to be simulated. This expansion is not included in the parametric studies introduced in Section 3 [26]. The equivalent volume is calculated accordingly:

(4) $$\begin{array}{c} V_{eq}=\frac{4}{3}\pi {\left(\frac{D_{eq}}{2}\right)}^{3} \end{array}$$

At low magnetic field strengths, the magnetization is not saturated. The following function was used to calculate magnetization at low magnetic fields [29]:

(5) $$\begin{array}{c} M_{m}=1+{19\left(10B\right)}^{0.16} \end{array}$$

where ***B*** is the magnetic field. The magnetic field also affects the chain length *n*, used to calculate the equivalent volume *V_eq_*. Both magnetization *M_m_* and n are therefore adjusted within reasonable recorded limits according to changes in the magnetic field ***B***.

The formula for the drag force experienced by the particles is based on Stokes law [30]:

(6) $$\begin{array}{c} F_{drag}=-3\pi \eta d\left(v_{p}-v_{f}\right) \end{array}$$

where *η* is the fluid viscosity, *d* is the average particle diameter, and *ν_p_* and *ν_f_* are the velocities for the particle and fluid, respectively.

The efficiency of the simulator can be improved by assuming a constant particle velocity within each discrete MiL timestep. When particles experience a change in force, such as through user input, it is also assumed that the experienced acceleration is sufficient to increase particle velocity to the point of the drag force balancing out other forces, all within one MiL timestep. These semi-static assumptions remove the requirement for calculating acceleration, reducing the complexity of the simulator. Equation (6) is instead used to directly calculate the particle velocity, where the drag force is substituted with the sum of all other forces acting on the particles. This occurs in every MiL.

**Figure 4:**
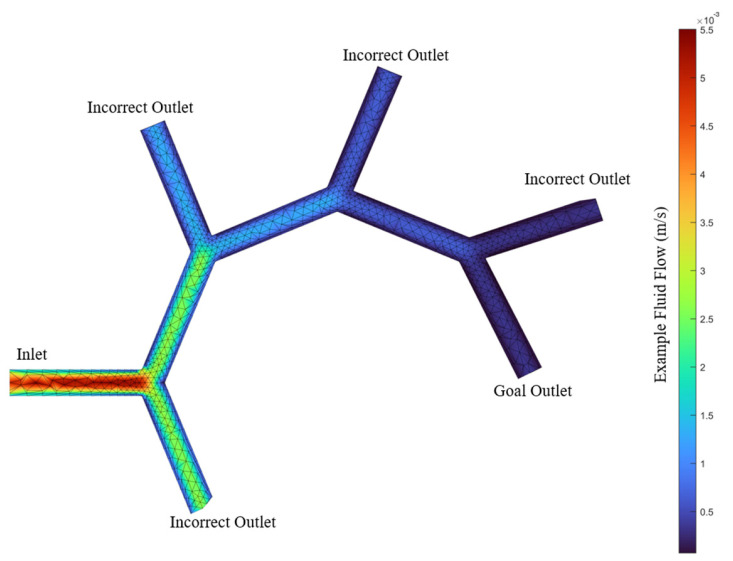
The fluid flow model simulated in COMSOL for the multi-bifurcation vascular network model.

In this research, a two-dimensional model using COMSOL Multiphysics (COMSOL version 6.1) was developed to simulate fluid flows ranging from 0.005 m/s to 0.06 m/s. The fluid flow at the inlet was given a central velocity of 0.005 m/s to 0.06 m/s. The selected fluid property in COMSOL was water (for simplicity). The default physical properties (such as dynamic viscosity and density) are implemented within COMSOL as a function of temperature, which is provided in Table 2. The dimension of the individual multi-bifurcations in COMSOL is 1 mm diameter for all branches. This is comparable to capillaries with similar range. Similarly, the chosen fluid flow rates are similar to relevant blood flow velocities [31]. The flow profile is shown in Figure 4. The multi-bifurcation was modelled in COMSOL as 3D tubes, but only the flow velocities along the plane in the centre of the pipes were used.

A COMSOL simulation utilizing the finite element method was used to generate data detailing the fluid velocity at each element for precise analysis. To integrate this model with the MATLAB simulator platform, the mesh (illustrated in Figure 4) was subsampled to align with the simulator’s structure. The structure of the multi-bifurcation shown in the simulator is representative of a simplified vascular network. The simulator is functional in 2D under the assumption that the simulated plane lies on the centrelines of all modelled vessels. Due to this, extending the simulator to three dimensions (with circular vasculature only) within the existing codebase is possible, but would introduce much greater complexity to users for the presented interface.

To efficiently retrieve fluid flow velocities, a lookup table correlating spatial positions within the simulated vascular structure to specific fluid flow velocities was constructed using the data recorded from the COMSOL model. This lookup table, which is loaded at runtime, significantly enhances the model’s efficiency without additional computational cost. It contains fluid-velocity data for approximately 7000 distinct locations, offering a detailed overview of fluid velocity.

Collisions between particles and the wall are modelled to be elastic. In the case of a collision, the component of the velocity orthogonal to the contact surface is mirrored such that the particle ‘bounces’. While in contact with the wall, particles have frictional force applied to them. To decrease the computation cost, other forces (***F****_other*) are neglected. Particle size influences both magnetic and drag forces (as can be seen in Equations (2) and (6)), and the platform is designed to adapt to these variations.

### 2.3. Experimental Study to Validate Simulator

**Figure 5:**
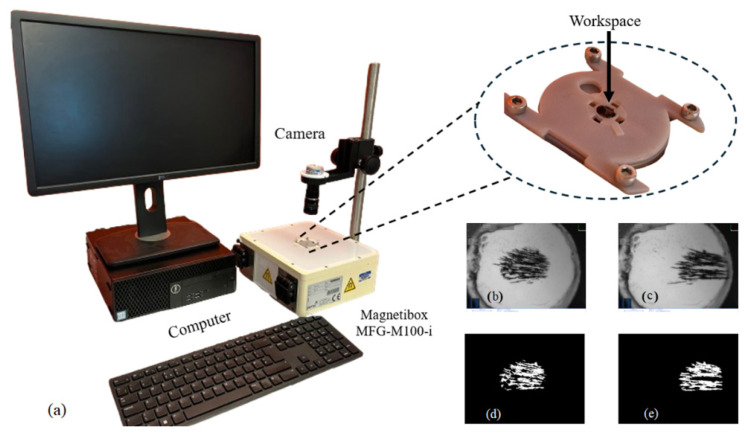
(**a**) Experimental setup showing the 3D-printed workspace in place on the MFG100 with optical camera. In the bottom-right corner, we show examples of (**b**) a raw start frame, (**c**) a raw end frame, (**d**) a pre-processed start frame, and (**e**) a pre-processed end frame. The preprocessing consisted of the segmentation of the particles from the background.

The mathematical model presented in Section 2.2 was verified using experimental data. A digital shadow approach was employed to replicate particle movement behaviour and validate it against the experimental settings. Fe_3_O_4_ un-coated nanoparticles (product number: 637106) were purchased from Sigma-Aldrich (3050 Spruce Street, St. Louis, MO, USA). The nanoparticles have a diameter between 50 and 100 nm. A concentration of nanoparticles suspended in water was created using 0.04 g of the nanoparticles and 83.2 mL of water to make a 2.08 g/L resultant suspension. Then, 30 µL of the resultant suspension was pipetted into the workspace displayed in Figure 5.

The workspace comprises a 3D-printed well and a 6 mm diameter glass slide affixed in the well using cyanoacrylate. The substrate provides a flat surface for particles to move smoothly. This workspace was affixed within the working area of an MFG-100-i system purchased from Magnebotix (Magnebotix AG, Ruetistrasse 14, 8952 Schlieren, Switzerland). The magnetic field generated over the whole workspace is assumed to maintain a constant gradient.

Each test consisted of resetting the workspace by concentrating the particles at the centre of the workspace with a permanent magnet, then inputting the experiment conditions to the MFG-100-i software. When the test was correctly set up, the MFG-100-i could be powered up, applying the magnetic gradient across the workspace to move the particles (demonstrated in Supplementary Video S1). Each test was recorded as a video file for later processing. The suspension was replaced with another 30 µL every 30 tests.

**Figure 6:**
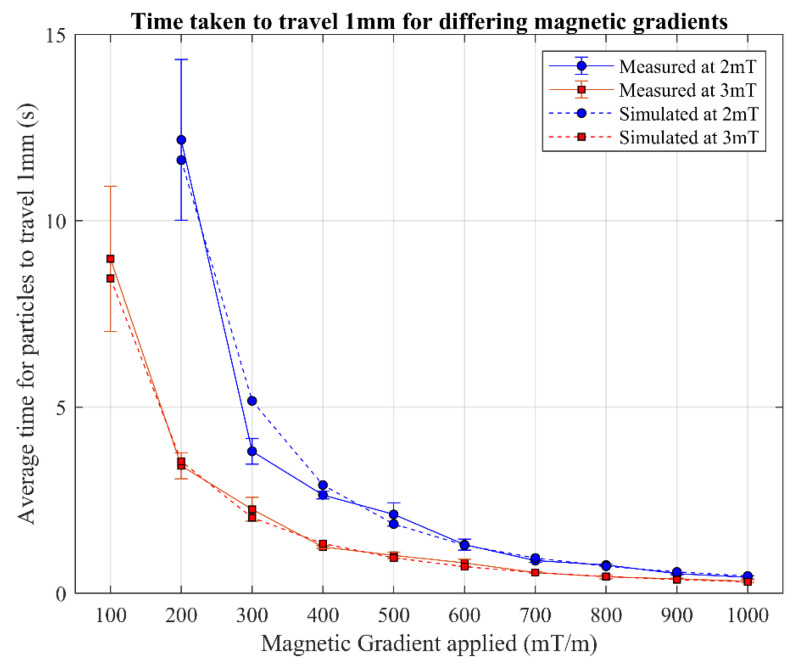
The time for particles to travel 1 mm at different magnetic gradients. For a magnetic field of 2 mT, a gradient of 100 mT/m did not produce a result (so is not present in the graph). The average of all shown deviations for each pair of simulation and real-world results is 8%.

The video files were binarized in a pre-processing step to isolate the particles in the images from the background, as shown in Figure 5. These binarized images were used to calculate the distance between the centres of mass of the selected start and end frames. The time between the frames was also recorded. This information was then used to calculate the average time taken to travel 1 mm, which was averaged over 4 tests for each parameter value investigated. The results of the experiments are shown in Figure 6.

The figure shows the experimental data compared to the simulated result, both obtained under the same conditions (same magnetic field and same travelled distance). The simulated results were also obtained under controlled conditions. The data show the time it took for the particles in a specified magnetic gradient to travel 1 mm. The average percentage difference across all reported datapoints presented in Figure 6 between the simulated and experimental data for a magnetic field of 2 mT is 10.4% and for a magnetic field of 3 mT is 5.9%. When averaged across both magnetic field strengths, an average variation of 8% is obtained. At a magnetic gradient of 100 mT/m within a 2 mT magnetic field, in the experimental study, the particles remained stationary.

## 3. Results and Discussion

Parametric studies were conducted on the platform to investigate the impact of various parameters on users’ ability to steer the swarm towards the goal outlet, thereby demonstrating the platform’s performance capabilities. The simulation parameters are presented in Table 3.

In this parametric study, an experienced user was asked to guide as many particles as they could to the goal outlet (shown in Figure 2). The goal of this parametric experiment was to provide a baseline for what performance an experienced user may obtain across different parameters, and which of these parameters might be interesting to investigate further with a larger participant cohort. The overall performance was measured as the percentage of 500 particles that reached the goal outlet, averaged over 10 tests per parameter. The error bars presented on the graphs represent the standard deviation over 10 tests undertaken per data point.

**Figure 7:**
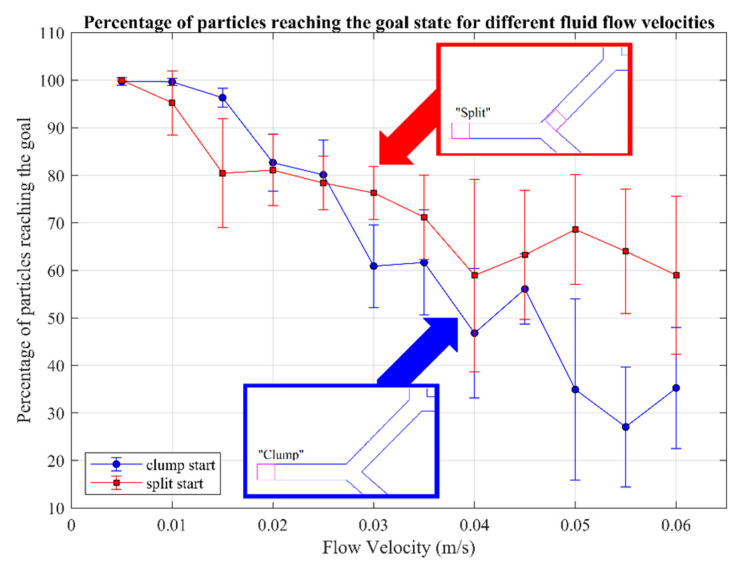
The percentage of particles to reach the selected goal outlet for a range of flow velocities. The split and clump labels refer to the starting configurations of particles, where clumped particles are positioned all in the same location, and split particles are positioned with equal distribution across two locations.

The first parameter investigated was the fluid flow (range shown in Table 2). In Figure 7, the blue line is labelled “clump start”. Clump here indicates that all particles start in the same area (randomized within the square drawn in magenta in Figure 7) as one collection of particles, and is the default starting positioning of the particles for all studies. The user was limited to 500 mT/m input. At 0.005 m/s flow velocity, 99.7% of particles reached the goal on average, with a low standard deviation represented as an error bar of 0.8%. This result then drops as the fluid velocity increases up to a maximum of 0.06 m/s, where an average of 35.2% particles reached the goal outlet, with a standard deviation of 12.7%. At 0.055 m/s, an average of 27% of particles reached the goal state. The significant standard deviation increase towards greater velocities demonstrates that, at higher velocities, the control the user has over the particles is reduced, indicating that a small incorrect input may result in a worse performance.

Focusing now on the effects of the particles’ initial spatial arrangement, in Figure 7 we also report the results obtained with the split start configuration (labelled “split start”). In this configuration, the particles are divided equally between two start locations: half of the particles are positioned after the first bifurcation and the other half are positioned in the clump configuration. The two locations are defined visually in Figure 7 in magenta squares. Default values were used for the other parameters (e.g., the limit of 500 mT/m for user input).

The starting configurations are compared to each other in terms of the difference in the average number of particles reaching the goal outlet for each fluid velocity. At lower fluid velocities, where the user has good control over the particles, the split configuration has a slightly lower success rate than clump. Once fluid velocity increases beyond 0.025 m/s, however, the split configuration demonstrates a greater success rate than the clump configuration. This occurs because at higher flow velocities a larger fraction of particles located in the clump are lost to the downward branch of the first bifurcation. Because in the split configuration 50% of the particles start after the first bifurcation, these cannot be captured by the first downward branch, facilitating a shorter path to reach the goal outlet. Error bars illustrate the data’s spread, indicating variability, not inaccuracies. A few low results affect the average, causing a wider standard deviation, despite a high average performance. For example, at a fluid flow of 0.01 m/s, the average number of particles reaching the goal outlet was 95.2%, but the standard deviation of 6.8% places the maximum error bar higher than the logical maximum of 100%.

The standard deviation of the split start is consistently greater than the clump start. An average of 58.9% of particles reach the goal at the maximum fluid flow of 0.06 m/s: 10% more than the number of particles starting after the first bifurcation. In contrast, at 0.06 m/s, the clump start showed around 30% of particles reaching the goal outlet. It can be concluded that while the starting configuration affected the number of particles reaching the goal, this was likely due to the reduced number of bifurcations the particles had to travel through. This effect is observed when the velocity of the fluid has a greater influence on particle movement than the magnetic gradient.

The platform allows the limiting of the gradient applied by the user, a continuous variable tested from 100 mT/m to 1000 mT/m, where 1000 mT/m is the maximum gradient that can be applied by the user in the simulator. This limit is implemented as a scaling of any input the user provides. This series of tests was performed with a fluid flow of 0.02 mm/s at a simulated magnetic field of 3 mT with a range of 100 mT/m to 1000 mT/m, as shown in Table 2.

**Figure 8:**
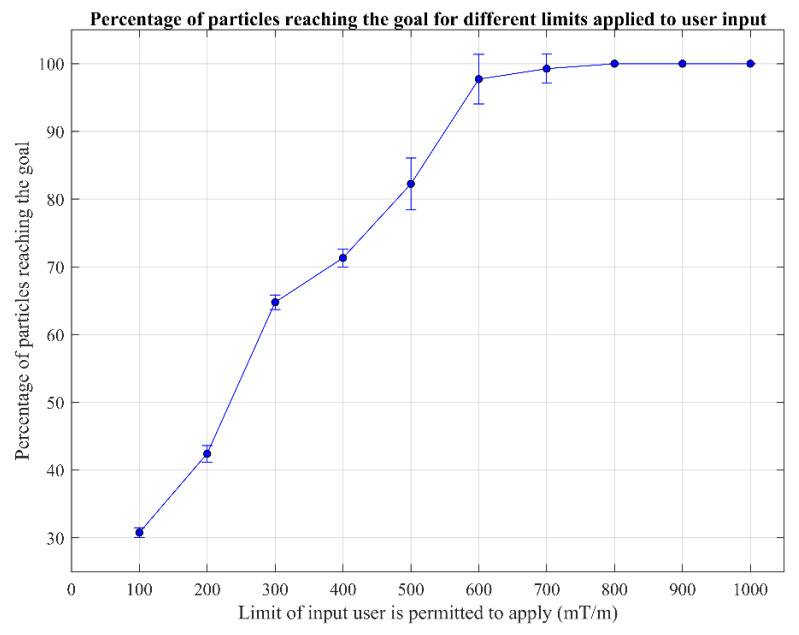
The percentage of particles to reach the selected goal outlet when the magnetic gradient is limited. At user input limit values of 800, 900, and 1000 mT/m, error bars are not present, as all recorded values were 100%.

Limiting the magnetic gradient had a large impact on the user’s ability to control the particle swarm. Figure 8 shows that for steeper gradients between 800 mT/m and 1000 mT/m, 100% of particles were directed to the correct output, with no recorded deviation. As no deviation was recorded, no error was recorded. Figure 8 displays the error bars (which represent the standard deviation) that sit above 100% at gradients of 600 mT/m and 700 mT/m, with high average success rates of 97.7% and 99.2%. This is due to a small number of datapoints bringing down the average from 100%, but does not imply that the values recorded were above 100% (graphed error bars indicate variability, not inaccuracies). The data below 600 mT/m trend downwards with a decrease in the gradient limit. This result is expected, as with a reduction in maximum gradient the user is able to apply less force onto the particles, so has less control over the trajectory of the particles in the channel. The lowest average recorded was 30.8% at 100 mT/m (at a magnetic field of 3 mT), with a low standard deviation of 0.7%. The maximum standard deviation was recorded at 500 mT/m as 3.8%, though this is not statistically significantly greater than at any other point, implying the cause was just discrepancies in user input.

## 4. Conclusions and Future Work

This paper introduces a real-time simulator platform with haptic input for microswarm steering. The verification demonstrated that the model is accurate, having an average deviation of 8% from the experimental data. The results show that users can control a simulated magnetic gradient field with a falcon haptic device to steer the microswarm over a range of COMSOL-modelled fluid-velocity profiles. The combination of these technologies has produced a responsive platform.

The effect of fluid flow on the control of the particles was investigated first, with a drop from 100% to 35.2% of particles reaching the goal when flow velocities increased between 0.005 m/s and 0.06 m/s. The final percentage of particles reaching the goal for the split starting arrangement at 0.06 m/s was 59%, implying that the starting particle configuration affected the outcome. Limiting the user input caused a drop in particles reaching the goal from 100% to 30.8%, with a change in magnetic gradient limit of 1000 mT/m to 100 mT/m (simulated at a magnetic field of 3 mT).

This parametric study demonstrated how different parameters affect users’ performance for an experienced user. In future works, this can be expanded to investigate how a cohort of participants who are not experienced with the simulator might perform compared to an expert user, or how this might represent the learning curve of the simulator. Future work could also extend the simulator to function as a fully integrated digital twin. The platform can be made adaptable for a variety of particle parameters, such as different particle sizes, enabling modifications to other parameters as needed.

This work presents a two-dimensional simulator to showcase the real-time haptic-based steering of a microswarm. A 3D simulator may be built upon multiple two-dimensional visual representations that concurrently show the particles’ locations in different planes. We regard this progression to a 3D framework as another possible extension of our current work.

## Figures and Tables

**Table 1:** Simulation parameters used in the 2D steering platform for the microswarm.

Parameter	Symbol Used	Simulation Value
Magnetic field (mT)	***B***	3
Permeability of free space (N/A^2^)	*µ*_0_	4π × 10^−7^
Magnetization (emu/g)	*M_m_*	33.74
Magnetic gradient (mT/m)	∇***H***	1000
Equivalent volume (m^3^)	*V_eq_*	7.95 × 10^−18^
Actual particle diameter (m)	*d*	75 × 10^−9^
Actual particle density (kg/m^3^)	*ρ*	4800
Average chain length (particles)	*n*	12,000
Equivalent diameter (m)	*D_eq_*	2.48 × 10^−6^
Fluid flow (m/s)	***ν*** *f*	0.005
Particle velocity(m/s)	***ν*** *p*	-
Fluid viscosity (Pa.S)	*η*	0.001
Number of particles	*N*	500

**Table 2:** COMSOL simulation parameters compared to real-world parameters.

Parameter	Range
COMSOL fluid flow (m/s)	0.005–0.06
Real-world fluid flow (m/s)	0.015–0.071
COMSOL vessel diameter (m)	0.001
Real-world vessel diameter (m)	0.0008–0.0018
COMSOL temperature (K)	293.15

**Table 3:** Parametric study parameters.

Parameter	Default Value	Variation Range
Magnetic gradient (mT/m)	500	100–1000
Limit of magnetic gradient (mT/m)	500	100–1000
Fluid flow (m/s)	0.005	0.005–0.06

## Data Availability

The original contributions presented in the study are included in the article; further inquiries can be directed to the corresponding author.

## Supplementary Materials

The following supporting information can be downloaded at: https://www.mdpi.com/article/10.3390/nano14231917/s1; Video S1: Recorded and simulated particle movement.
